# Modeling the distribution of jet fuel price returns based on fat-tail stable Paretian distribution

**DOI:** 10.1371/journal.pone.0309975

**Published:** 2024-10-30

**Authors:** Shuang Lin, Shengda Zhang, Chaofeng Wang, Fan He, Zhizhen Xu, Yuchen Zhang

**Affiliations:** 1 School of Economics and Management, Civil Aviation Flight University of China, Deyang, China; 2 School of Airport Engineering, Civil Aviation Flight University of China, Deyang, China; 3 School of Transportation Science and Engineering, Beihang University, Beijing, China; 4 Department of administration, Chengdu Industrial Research Institute Branch of China Mobile Communication Group Co Ltd, Chengdu, China; Aalto University, FINLAND

## Abstract

Jet fuel plays a crucial role as an essential energy source in aerospace and aviation operations. The recent increase in fuel prices has presented airlines with the new challenge of managing jet fuel costs to ensure consistent cash flow and minimize operational uncertainties. The conventional risk prediction models used by airlines often assume that risks are normally distributed according to the classical Central Limit Theorem, which can lead to under-hedging. This paper proposes an innovative approach using the stable Paretian model to analyze the price return of jet fuel in large samples. It comprehensively compares the fitting effect of the stable Paretian distribution with that of the normal distribution based on specific criteria and non-parametric significance tests. Furthermore, it investigates the accuracy of risk measures such as Value at Risk (VaR) and Conditional Value at Risk (CVaR) predicted by both models. In addition to comparing differences in VaR between predicted values and actual values, this paper provides a more comprehensive comparison of risk measures under rolling window forecast situation. Results suggest that despite indistinguishable results in VaR backtest, the stable Paretian distribution has a overall better fitting effect as well as a less biased predicted CVaR based on the AIC of -14099.46, BIC of -14110.98, *p* = 0.58 in Kolmogorov-Smirnov test and *p* = 0.46(0.92) in the 0.01(0.05) significance level of Expected Shortfall Regression Test. This might be explained by its ability to capture asset return dynamics while maintaining shape stability with few parameters. This research can provide valuable insights for guiding airlines’ risk management decisions. its ability to capture asset return dynamics while maintaining shape stability with few parameters. This research can provide valuable insights for guiding airlines’ risk management decisions.

## 1. Introduction

**Fig 1:**
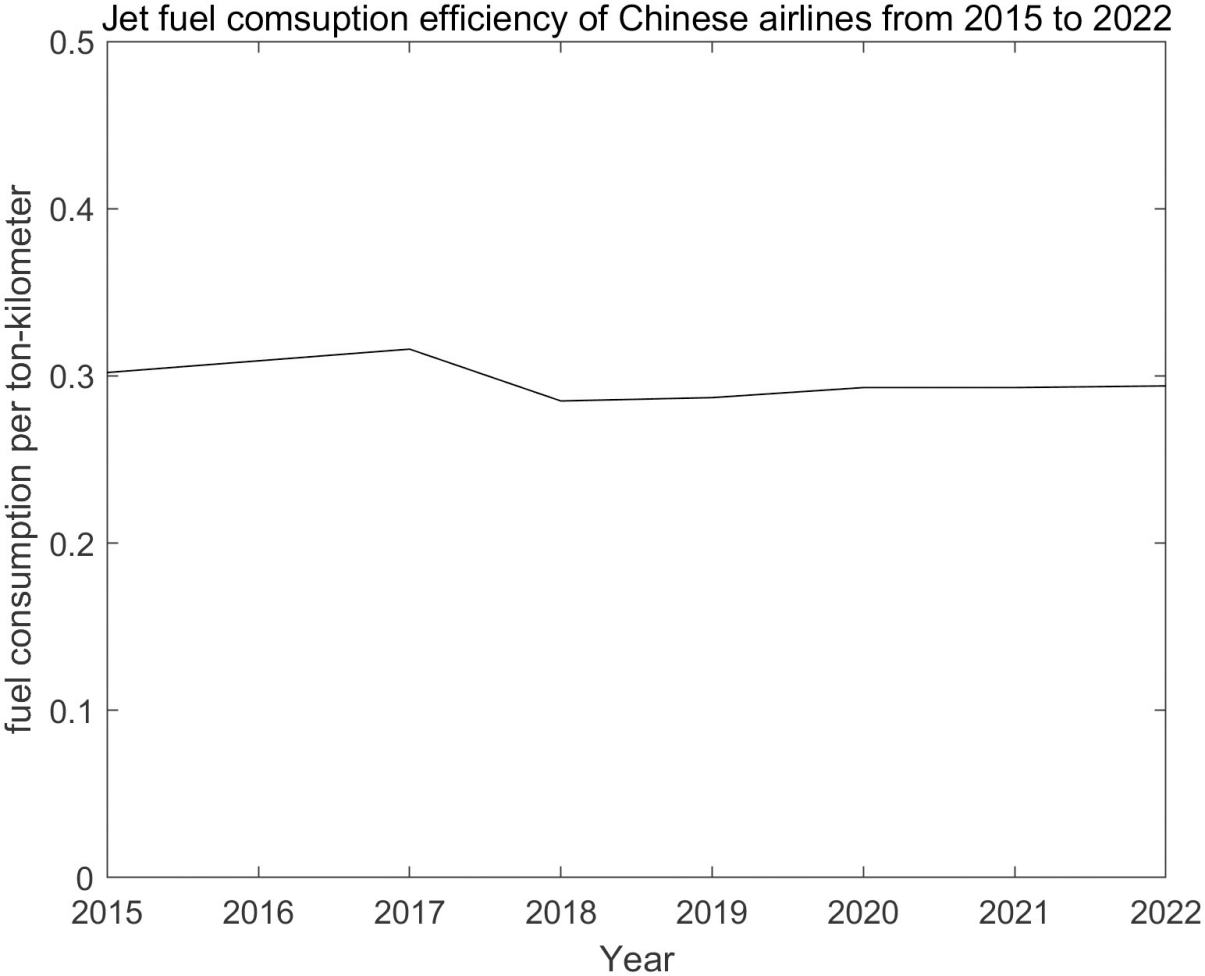
The jet fuel consumption efficiency of Chinese airlines.

Fuel procurement costs account for a large part of the major costs of airlines [1]. Keeping jet fuel costs less volatile or more predictable reduces cash flow uncertainty, which is good for financing. Therefore, large airlines usually take corresponding measures to hedge the price risk of jet fuel [2]. Specifically, for a given transportation demand, if the purchase volume is treated as an exogenous variable, then the annual jet fuel cost is determined by the purchase volume multiplied by the purchase price per gallon, so stabilizing the purchase price is key to reducing fuel cost volatility. Indeed, the profits of large airlines are typically very sensitive to changes in fuel prices. In the case of China Southern, Asia’s largest airline by passenger traffic for more than four decades, every 1% increase in fuel prices last year reduced profits by more than 2%. The reason is not only that jet fuel, as the primary energy source, is irreplaceable under current technological conditions, but also that the means to reduce consumption are very limited [3]. The jet fuel consumption efficiency of Chinese airlines in the past few years has remained relatively stable at about 0.3 kg per ton-km, as shown in Fig 1. Therefore, the implementation of risk management to reduce price volatility seems to be the most sustainable mechanism at present. Scholars have proposed various hedging strategies to stabilize the fuel cost [4–6]. However, research focusing on the return distribution of jet fuel is rare. Despite the price movement of some types of energy tends to correlate with each other such as jet fuel with crude oil, price variation of jet fuel can be special when the price of crude oil surges or intensive military operations emerge. Therefore, studying the price movement of jet fuel instead of another type of energy is necessary [7]. Compared with crude oil returns, the distribution of return of jet fuel prices is more likely to show fat-tail as well as asymmetry features, which might be explained by characteristics of concentrated buyer and seller as well as way of dealing, i.e. transaction of jet fuel usually happens over the counter [3]. Since airlines are so sensitive to the tail risk of return of jet fuel, the application of a proper probability model that captures statistical features including fat tail, asymmetry as well as maintaining shape stability [8] is necessary based on the logic of Generalized Central Limit Theorem and efficient market hypothesis. Thus part of the challenges to reach this goal is to observe the effectiveness of a stable paretian distribution model and conduct a comprehensive test for both fitting effect and forecast precision. By employing different goodness-of-fit tools along with risk-measure testing methods, we hope to overcome these challenges.

The initial step in effectively hedging the risk of jet fuel is to identify and calculate the risk that needs to be hedged. However, when airlines calculate price risk, most of them utilize traditional risk models which heavily rely on the assumption that the random variables, such as asset returns (interpreted as percentage changes in price), adhere to a normal distribution. Nevertheless, numerous empirical studies have concluded that asset returns frequently deviate from a normal distribution due to their leptokurtic, fatty-tailed, and asymmetric marginal distributions. In such cases, using usually distributed returns may result in underestimating the risk. Researchers have identified various probability distribution models—such as generalized hyperbolic, generalized asymmetric t (gat), extreme value or Laplace distribution—to describe non-normal patterns of return rates. However, as revealed by Calzolari et al., they either employ a greater number of parameters or lack shape stability when subjected to summation [8]. Furthermore, their grounded theorem lacks the strength of the Generalized Central Limit Theorem. Therefore, this study exclusively focuses on the stable Paretian probability model and utilizes it to characterize the stochastic process of return rates of jet fuel in comparison with the common Gaussian probability distribution [2, 3]. The utilization of a stable probability distribution family is prevalent in academic research due to its robust theoretical support, favorable mathematical properties with minimal parameters, and substantial empirical evidence [9–12]. The stable Paretian distribution, as a unique type of stable probability distribution family, has garnered significant interest from both theoretical and empirical researchers [13–15]. We do not assert that the stable Paretian distribution correctly captures patterns of fuel returns, as we believe no model can. However, despite its drawback of infinite variance, the stable Paretian distribution is supported by solid theorems, has few parameters, and possesses proper statistical properties that capture features such as asymmetry and fatty tails. This justifies its rationality for computing price risk and ultimately benefits corresponding risk management decisions.

Previous researchers have extensively explored the theoretical aspects of stable probability models. However, empirical studies on calculating price risk using stable distribution have been relatively rare until recent decades. This is mainly due to the lack of explicit expressions in most stable distributions and their infinite variance statistical properties, which require extensive calculations for large sample data and heavy reliance on computer technology. With recent advancements in theoretical research on stable probability models and the development of computer technology, empirical research based on stable distribution has started to emerge. The concept of stable distribution (also known as alpha-stable distribution) was first introduced by Levy in 1925 while he was investigating the properties of the sum of independent and identically distributed random variables. Nolan furthered the research based on his predecessors by establishing the characteristic equation for the stable distribution and providing a method for parameter estimation via asymptotic expression. This work lays a solid foundation for subsequent scholars to utilize the stable probability model [16]. Weidong Xu et al. have shown the superior descriptive capability of stable distribution in characterizing Chinese stock returns, as compared to the renowned Black-Scholes model [17]. Nolan advocated for the utilization of stable probability models in price dynamics due to their superior fit to data across a wide range. In contrast, normal models inadequately capture the probability density of empirical data, particularly by underestimating the likelihood of extreme values or outliers. The adoption of stable models holds significant potential for enhancing price risk management and insurance actuarial practices [18]. Royuela-del-Val innovatively programmed a superior algorithm which dramatically benefit the application of it in cognitive computing [19]. Masaru Shintani and Ken Umeno gave intuitive proof that, as long as we regard logarithmic returns of stock as independent random variables with the same power-law index α, summation of returns would converge to stable distribution even those random variables come from non-identical distribution [20]. Liu applied stable model to predict factors affecting roadway surrounding rock stability [21]. Samet et al. conducted their empirical research to model volatility of three types of energy futures through two time series models based on three different distribution assumptions. They concluded that APARCH model fits sample time series better in most cases under general extreme value distribution (GED) while GARCH model slightly outperformed under stable distribution. At the same time, risk measurements in the energy market tend to be significantly underestimated based on conventional models instead of that based on APARCH model and general extreme value distribution [22]. Liu Kwangil Bae found out that stable distribution is the possible reason for short-term momentum and long-term reversal [23]. Nolan illustrated the new numerical integration method to compute cumulative distribution functions(CDF) of the stable probability model thus further benefit the application of stable distribution in practice [24]. Tong Liu et al. combined the temper stable distribution and the CGARCH model to model volatility of asset returns via simulations and empirical data from Shanghai Stock Exchange. Both results indicate that CGARCH model with assumption that random variables follow a tempered stable distribution work better compared to CGARCH based on other distributional assumptions [25]. Andrei et al. employed the stable distribution for optimal portfolio construction by buying S&P500 sector ETFs, as a result they successfully developed an investment strategy which significantly outperform S&P500 index [26]. Meintanis validated data fitting effect of multivariate stable Paretian law through various goodness-of-fit tests [27].

As for work related to computation of energy risk, plenty of researches have been done recently. Muteba et al. found evidence of a structure break in the volatility process as well as leverage effects in data-driven research covering BRICS stocks and world crude oil prices [28]. Zhou et al. identified the volatility spillover effect in renewable energy market based on BEKK-GARCH and multidimensional analysis [29]. Samunderu and Murahwa found a more accurate computation for risk of bond index via combining EGRCH and a non-normality presumption [30]. Lin et al. attempts CVaR and MS-GARCH to model price risk of aviation fuel, which as a result effectively stabilized the volatility of fuel cost [6]. Jing adopted Asymmetric Laplace Distribution to effectively compute VaR and CVaR [31]. Yanto introduced Cluster-Based model to improve Efficiency of Fuel Budgeting [32]. Cao suggested a Multivariate GARCH to predict risk of jet fuel and implement composite associated hedging strategy [5]. Tang employed the Bayesian DCC-MGARCH and frequency connectedness methods to model risk among the green bond, clean energy, and fossil fuel markets [33]. Samunderu proposed a two-tier model to compute Jet Fuel Price Risk and proved it a valuable method to guide airlines’ risk management strategies [34].

Since the application of stable Paretian distribution to calculate potential future losses in jet fuel positions and the investigation of the accuracy of these calculations for sustainable airline management have not yet been thoroughly studied, this research aims to address this gap by conducting empirical research rather than solely making theoretical contributions. We aim to contribute to risk management by following design. First, we compare the goodness-of-fit result from the stable Paretion model and Gaussian model using information ratio, KS test, and stabilized probability plot, all results show an outperformance from Paretian model. Then we evaluate the accuracy of predicted risk measures using rolling window forecasts to obtain a series of out-of-sample prediction results, the discrepancies between predictions and actual losses are carefully observed and tested via both interval forecast coverage testing and regression test. While the prediction model shows no superiority in VaR testing, it gains a positive result from CVaR regression testing under 1% alpha level, which suggests a better effectiveness of the prediction model especially under extreme conditions.

## 2. Methodology

In this section, we offer a comprehensive explanation of the probability distribution used in our analysis to model logarithm returns of jet fuel and subsequently evaluate price risk. Additionally, we delve into the definition of probability distribution, conduct a goodness-of-fit test, and present various risk measures as part of our methodology.

### 2.1 Stable Paretian distribution

#### 2.1.1 Definition

Stable distributions, also known as alpha stable or Lévy stable distributions, constitute a class of probability density distributions with valuable mathematical properties that can effectively capture asymmetry, leptokurtic, and fat-tail regularities using only 4 parameters in addition to shape stability. In the context of this paper, the term "stable Paretian" specifically denotes non-Gaussian stable distributions, excluding the normal distribution which can be regarded as a special case of alpha stable distribution. The term "stable" signifies that the shape of X remains unchanged (only scaled and shifted) under addition. Similarly, the term "Paretian" is employed because tails of non-Gaussian stable distributions demonstrate a power-law behavior or asymptotically adhere to Paretian law. Various definitions for stable distribution exist due to prior research; therefore, we adopt the definition based on shape stability and characteristic function provided by John P. Nolan (1997) [16].

In shape stability definition, we define non-degenerate random variable(r.v.)*X* as stable there are real numbers *c*_*n*_ > 0 in addition to *d*_*n*_ ∈ *R*, if for all *n* > 1 such that

$$X_{1}+X_{2}+\cdots +X_{n}≝c_{n}X_{i}+d_{n}$$ (1)

Where *X*_1_, *X*_2_,…,*X*_n_ are i.i.d copies of *X*. When *d*_*n*_ = 0 for all n we define X as strictly stable, otherwise as quasi-stable, which is often the case including this work. As long as r.v. is quasi-stable, linear transformation would not change shape of its probability density distribution.

In characteristic function definition, the distribution of a random variable *X* is defined as stable and notates *X* ~ *S*(α, s defiδ_1_; 1) if it has characteristic function—the Fourier transform of probability density function below:

$$E\left(e^{iuX}\right)=\left\{\begin{array}{c} e^{\left(-\gamma^{\alpha }{\left|u\right|}^{\alpha }\left(1-i\beta \;\text{tan}\left(\frac{\pi \alpha }{2}\right)\left[sign\left(u\right)\right]+i\delta u\right)\right)},\;\alpha \neq 1\\ \;\\ e^{\left(-\gamma \left|u\right|\left(1+i\beta \frac{2}{\pi }\left[sign\left(u\right)\text{log}\left|u\right|\right]\right)+i\delta u\right)}\;,\;\alpha =1 \end{array}\right.$$ (2)

Where 0 < *α* ≤ 2, −1 ≤ *β* ≤ 1, *γ* ≥ 0, *δ* ∈ R, *u* ∈ *R*, $i=\sqrt{-1}$, notice that first 4 Greeks are shape parameters. The first shape parameter *α* describes tail behavior, the second shape parameter *β* shows the skewness of the distribution. The third and fourth shape parameter *γ* and δ_1_ describe scale and location respectivelywhere δ_1_ is the expectation of population probability density distribution. Notice that when *α* = 2 the stable distribution becomes normal distribution, so the term “stable distribution” in following sections refers in particular to stable Paretian distribution(sometimes called non-Gaussian distribution)with the tail parameter *α* < 2.

The last integer number 1 denotes a certain way of parameterization considering there are over 10 ways of parameterization for stable probability family favored by different researchers at the mean time *δ* is defined differently under different parameterization. In fact, these diverse parameterizations had caused tremendous confusion which possibly tempered the application of it in empirical analysis. In some literature, researchers employed the notation *S*(*σ*, *β*, *μ*) for the class of stable laws, others adopted the notation *S*(*α*, *β*, 1). Considering those parameterizations are related to combination of historical evolution as well as mathematical analyze methods, here we decide to take the first modified parameterization notation brought up by Nolan as X ~ S(*α*, *β*, *γ*, *δ*_1_; 1) for following reasons. Usually, Greek *α* in distribution parameter is treated as different and fixed. Nonetheless all four parameters *α*, *β*, *γ*, *δ* are unknown and need to be estimated in empirical study of statistical reference, so the aforementioned notation expressed this idea clearly. Additionally, the scale parameter *γ* andlocation parameter *δ* iscommonly mistaken as standard deviation *σ* and expectation *μ*, which are not always true even in the normal probability distribution, so instead of adopting symbols *σ* for the scale and *μ* for the location, we take *γ* and *δ*. Lastly, the integer1 is used to distinguish among various parameterizations in which *δ* possibly means different. We believe everyone who adopt stable probability density distribution model should clearly state which parameterization is taken so that some misunderstanding could be avoid.

The advantage of this form X ~ S(*α*, *β*, *γ*, *δ*_1_; 1) includes the nice algebraic features along with simplicity of characteristic function, both of which leads to preferability. However, one might choose to employ other parameterizations if other needs are required like continuity everywhere.

Please note that for most stable distributions, there is no explicit closed formula available, with the exception of a few special forms such as the normal, Cauchy, and Lévy distributions. It is challenging to directly derive Value at Risk (VaR) and Conditional Value at Risk (CVaR) by integrating the density function; therefore, in the following experiment, we utilize random variable simulation to obtain predicted risk measures.

#### 2.1.2 Parameter estimation

There are several ways to estimate 4 parameters of certain stable distribution, In this paper we employ maximum likelihood (ML) estimation algorithm in Matlab software given by Nolan [16], however, the basic idea is the not much different as ML estimation in other model.

For a vector of observations *X* = (*X*_1_ + *X*_2_ + ⋯ + *X*_*n*_), the ML estimation of the parameter vector *θ* = (the ML esti is obtained by maximizing the log-likelihood function, define the log-likelihood function:

$$l\left(\alpha ,\;\text{s}\;\text{obtai}\right)=\sum_{\text{i}=1}^{\text{n}}ln\;\tilde{f}(X_{i}|\alpha ,\;\text{n}\;\text{ntai)}$$ (3)

The tilde denotes that no explicit density function hence we have to approximate it via judiciously chosen methods involving embedding characteristic function in the complex plane and employing contour integration. Since our work focus on empirical application instead of making contribution to theoretical field, elaboration of mathematics derivation is beyond our scope here.

#### 2.1.3 Generalized Central Limit Theorem (GCLT)

Generalized Central Limit Theorem (GCLT) claims that the appropriate normalized sum of i.i.d random variables will converge to a stable distribution even for variables with infinite variance. Formal definition of GCLT could be found in work of Chen [35]. Recently Masaru Shintani and Ken Umeno further gave some proof that summation of asset logarithmic returns would converge to stable Paretian distribution even those random variables come from non-identical distributions as long as those distributions have same first shape parameter α [8].

In order to comprehend the rationale behind applying GCLT to model return rates of price data, it is imperative to assume the efficiency of the jet fuel market. According to the efficient market hypothesis, despite potential irrational behavior exhibited by individuals, the market as a whole accurately prices assets based on all available information. Consequently, changes in asset prices are indicative of new information rather than irrational behavior from any individual investor. Considering the diverse array of market participants and complex composition, the arrival of new information can be viewed as continuous over time, akin to fluctuations in price. Therefore, the distribution of fuel returns essentially represents the dissemination of novel information. If we consider a piece of new information randomly extracted from the information set within a small-time interval as an independent identically distributed (i.i.d) random variable, then the daily price return is the cumulative impact of all the new information on a given day; ultimately, the observed daily asset return is the aggregate result of numerous small components. According to GCLT, this aggregation converges to a stable distribution. Additionally, in a stochastic process based on a stable model, there are infinite numbers of jumps over any time interval, with most being small and approximating transitions in asset prices between neighboring decimalized prices. Those logics explain the rationality to model return rates of public traded asset with stable distribution, meanwhile as long as the first shape parameter α is not 2, which is true in most cases, this stable distribution can be called stable Paretian distribution.

While the assumption of complete independence of data is stringent, the efficient market hypothesis posits that in an efficient market for publicly traded assets, the current price incorporates all past information and expectations. Therefore, it becomes impossible to predict future price movements based on past data or intercorrelations among data. This is because if such predictions were feasible, individuals would act upon this information by buying when correlations indicate a future rise or selling when they do not. Consequently, asset prices would adjust towards a new equilibrium that reflects the correlations discovered by market participants. Furthermore, it is common to make assumptions about independent random variables in academic studies; for example, in transportation management, one often assumes that vehicle volume passing through a road or space during a certain time period follows specific models such as Poisson probability distribution for ease of computing sample statistics. However, a lower-than-anticipated number of vehicles during non-peak hours may be associated with a higher-than-expected number of vehicles during rush hour upon closer examination. In the realm of data mining, correlations can often be uncovered by delving deeply into the data. Therefore, for the sake of convenience and mathematical tractability, we make use of this assumption despite acknowledging its imperfections.

### 2.2 Goodness of fit test

#### 2.2.1 Kolmogorov-Smirnov test

In order to gather additional evidence supporting the superior performance of the stable Paretian probability model without relying on density formulas, we intend to conduct null hypothesis significance tests (NHST) for both sets of fitting results. Unfortunately, there is currently no specific NHST designed for the stable model. However, given that we are not making any underlying distributional assumptions about the data, nonparametric techniques are suitable choices for an unknown distribution. Considering that the application of a Chi-square test requires subjective partitioning to obtain different bins, which may result in varying outcomes based on how the data is grouped, we have opted for the versatile Kolmogorov-Smirnov (KS) test. This test has been proven to be applicable for all continuous distributions within our limited options.

KS test is a popular nonparametric NHST to test how well the empirical distribution function from actual data fit the theoretical distribution function based on the difference between their CDF, or to compare whether the distributions of two groups of samples are the same. The advantage of KS test is that it applies to all continuous distribution, so we employ this method to test whether the out of sample historical data fits the stable Paretian distribution or normal distribution. The null hypothesis and test statistic are:

$$H_{0}:S\left(x\right)={\text{F}}_{0}\left(x\right),\;\forall x;$$ (4)

$$H_{1}:S\left(x\right)\neq {\text{F}}_{0}\left(x\right),\exists x$$ (5)

$$S\left(x\right)=\frac{\#\{X_{i}<x,i=1,2,\ldots ,n\}}{n}$$ (6)

$$D_{n}={\text{max}}_{0\leq \text{i}\leq \text{n}}\{\text{max}(|\text{S}(x_{\text{i}})-{\text{F}}_{0}\left(x_{\text{i}}\right)|,|\text{S}(x_{\text{i}-1})1{\text{F}}_{0}\left(x_{\text{i}}\right)|\;)\}$$ (7)

S(x) = the empirical cumulative distribution function calculated from sample data, F_0_ (x) = the theoretical distribution. The test statistic *D*_*n*_ measures the maximum distance between the empirical and theoretical distributions, with *Dn* following the Kolmogorov distribution as n approaches infinity under the assumption that *H*_0_ is true. By comparing the test statistic *D*_*n*_ to the critical value *D*_*nα*_, we can draw conclusions regarding whether the empirical distribution significantly differs from the theoretical distribution given a large sample size.

#### 2.2.2 Stabilized probability plot

The stabilized probability plot represents an advancement from the traditional percent-percent (PP) plot and serves as a graphical goodness-of-fit test. The PP plot is commonly utilized to illustrate the distribution pattern of data, with the cumulative proportion of the sample on one axis. In contrast to QQ plots, the abscissa of data points in PP plots depend solely on sample size, preventing clustering of points. The stabilized probability plot employs a sine transformation to stabilize variances of plotted points, thereby enhancing interpretability by maintaining approximately equal variances. Additionally, acceptance regions can be conveniently identified through the use of two straight lines. Therefore, apart from the KS test, we also apply stabilized probability plot to visually show stable Paretian distribution behave more aptly in this work. The sample statistic *D*_*SP*_ which, analogous to the standard KS statistic *D*_*n*_, is also defined to be themaximum deviation of the plotted points from theoretical values. In fact, Michael employed distribution function of two-sided KS test to determine the critical value of *D*_*SP*_. The abscissa and ordinate of PP plot and stabilized probability plot arecomputed in Table 1.

**Table 1:** The abscissa and ordinate of PP plot and stabilized probability plot.

Methods	abscissa	ordinate
PP plot	$(\text{i}-\frac{1}{2})/\text{n}$	F_0_{(*x*_i_ − μ)/n}
stabilized probability plot	$\frac{2}{\pi }\text{arcsin}{\left\{\left(\text{i}-\frac{1}{2}\right)/\text{n}\right\}}^{\frac{1}{2}}$	$\frac{2}{\pi }\text{arcsin}\left\{{{\text{F}}_{0}}^{\frac{1}{2}}\left\{\left(x_{\text{i}}-\mu \right)/\text{c}\right\}\right\}$

Annotation: Where *x*_1_ ≤ *x*_2_ ≤ ⋯ ≤ *x*_n_ are an ordered random sample of size n, i is the sequence number of *x*_i_ in sorted sample. F_0_ is thetheoretical distribution. Moreover, the reference line issimply the diagonal which abscissa equals ordinate, two acceptance region line are parallel to reference line across distance of *D*_*SP*_, which is asymptoticallyequivalent to weighted KS statistic under certain condition.

### 2.3 Risk measures

#### 2.3.1 Value-at-risk(VaR)

Wall street institutions generally use value-at-risk VaR to measure the maximum possible loss value of assets during a specific time in the Future. In this work we only talk about the VaR expressing in rate of return, however it could be expressed in currency unit with more steps to compute. Supposing $X\in {\mathbb{R}}^{\text{n}}$ stands for assets, $S\in {\mathbb{R}}^{\text{n}}$ stands for uncertainties with the probability density function *p*(*S*), *x* and *s* are decision vectors, *f*(*x*, *s*) stands forloss of assetsfor corresponding assets and risk factors. Given any probability level *β*(0<*β*<1) and time period t, the cumulative probability distribution function *φ*(*x*, *α*) such thatupper limit of possible losses of asset is smaller or equal to *α* is given by:

$$\varphi \left(x,\alpha \right)=\int_{f\left(x,s\right)\leq \alpha }\;p\left(S\right)dS$$ (8)

For simplicity, assuming *p*(*S*) and *φ*(*x*, *α*) are continuous everywhere. Then *φ*(*x*, *α*) full determines the dynamic of thisr.v., we can express the corresponding VaR as:

$$\alpha_{\beta }\left(x\right)=min\left\{\alpha \in \mathbb{R}:\varphi \left(x,\alpha \right)\geq \beta \right\}$$ (9)

*α*_*β*_ is the = VaR under significance level *β*.

#### 2.3.2 Conditional Value-at-Risk (CVaR)

In practical applications, VaR exposes its limitations, including(a) a lack of subadditivity and convexity; (b) there may be multiple local minimum values when minimizing the problem, which does not meet the requirements of risk ‘consistency’; (c)gives no information for the left part of distribution below *α* quantile. Then comes another method of risk measurement: Conditional Value-at-Risk CVaR(sometimes called Expecting Shortfall). Similarly, given the confidence level 1e*β* and time period t, CVaR is the conditional expectation of the asset in condition that loss X is greater(in absolute value)than the corresponding VaR value *α*_*β*_(*x*), the formula can be denoted as follows:

$$\varphi_{\beta }\left(x\right)={\left(1-\beta \right)}^{-1}\int_{f\left(x,s\right)\geq \alpha_{\beta }\left(x\right)}\;f\left(x,s\right)p\left(S\right)dS$$ (10)

*φ*_*β*_(*x*) = defined as CVaR of assets under significance level *β*.

### 2.4 Evaluation of forecast risk measures

In order to better evaluate the effectiveness of predicted risk measures based on stable Paretian distribution, we utilize rolling window forecasts to generate a series of out-of-sample predictions for VaR and CVaR. Subsequently, we compare these risk predictions with actual losses to identify any discrepancies. Through these methods, we can assess whether forecasts derived from the new model are able to more accurately capture real losses or at least mitigate bias when unexpected severe risk events occur.

#### 2.4.1 Coverage test for VaR

For the VaR test, we choose the log-likelihood ratio of the unconditional coverage test given by Kupiec [36] and the log-likelihood ratio of the conditional coverage test proposed by Christoffersen [37]. Both unconditional and conditional coverage tests rely on the indicator sequence ${\left\{I_{t}\right\}}_{t=1}^{T}$ constructed from a given interval prediction used in dynamic situations. While the former focuses on average(unconditional) coverage, the latter is improved to detect whether the interval prediction considers clustered outliers because the prediction range should dynamically vary through time. If risk measures are correctly predicted, the test statistic log-likelihood ratio should follow a chi-square distribution. Actual loss that exceeds our prediction is also presented in the test to compare the performance of two models.

#### 2.4.2 Dynamic quantile test for VaR

We also utilize dynamic quantile(DQ) test designed by Eagle and Manganelli to check whether the probability of loss in every period exceeding the VaR is independent of past information [38]. This method models the quantile directly rather than modeling the whole distribution as VaR is tightly linked to the standard deviation of return distribution which should be autocorrelated during periods of volatility cluster. Therefore, DQ test constructed the conditional autoregressive value at risk (CAViaR) model which specifies the evolution of the quantile over time using an autoregressive process and estimates the parameters with regression quantile. A generic CAViaR specification is presented below:

$$f_{t}\left(\beta \right)=\;\beta_{0}+\beta_{1}f_{t-1}\left(\beta \right)+\beta_{1}l(x_{t-1})$$ (11)

where *f*_*t*_(*β*) denotes the *θ* quantile of the distribution of asset returns formed at time t, *β* is a vector of unknown parameters while *l* is a function of a finite number of lagged values of observables. The parameters are estimated by minimizing the regression quantile loss function, then a constructed test statistic following chi-square distribution is then computed to run significance test.

#### 2.4.3 Regression test for CVaR

For the CVaR test, we adopt the Expected Shortfall Regression Backtest (ESR) shed light on by Bayer and Dimitriadis [39]. This method can assess the correctness of forecasted CVaR risk measures. The advantage of this state-of-art backtest is that we can test with only forecasted CVaR one explanatory variable, which is quite convenient in contrast to other current backtest methods. The preliminary setup of ESR tests, also called auxiliary ESR test, is designed as a joint regression model for the VaR and CVaR. Regression models are presented as follows:

$$y_{t}=\;\beta_{1}+\beta_{2}\hat{v_{t}}+{u_{t}}^{q}\;and\;\text{and}\;y_{t}=\;\gamma_{1}+\gamma_{2}\hat{e_{t}}+{u_{t}}^{e}$$ (12)

$${\text{H}}_{0}\left(\gamma_{1},\gamma_{2}\right)=\left(0,1\right)\;\text{against}\;{\text{H}}_{1}\left(\gamma_{1},\gamma_{2}\right)\neq \left(0,1\right)$$ (13)

Where {***y***_***t***_} is the response data, $\hat{v_{t}}$ and $\hat{e_{t}}$ is predicted VaR and CVaR respectively. Under the asymptotic theory they proposed, these two variables are collinear such that Eq 11 is simplified as

$$y_{t}=\;\beta_{1}+\beta_{2}\hat{e_{t}}+{u_{t}}^{q}\;\text{and}\;\text{and}\;y_{t}=\;\gamma_{1}+\gamma_{2}\hat{e_{t}}+{u_{t}}^{e}$$ (14)

Eq 12 is also called the strict ESR test. Therefore, we only need return data as well as forecast CVaR to test whether there is any significant difference between predicted risk measures from two probability distributions. The test statistic is constructed as the Wald statistic and we implement the R package esback to complete corresponding procedures [39]. For properly forecasted CVaR, the intercept term should be zero, and the slope should be one, which means a failure to reject the null hypothesis.

## 3. Results and discussion

In this section, we begin by modeling the return rates of jet fuel using both stable Paretian distribution and normal distribution. The empirical analysis utilizes the daily logarithmic returns of Coast Kerosene-Type Jet Fuel Spot Price, which is available at the US Energy Information Administration from January 1, 2010 to June 30, 2023, encompassing over 3000 data points. The selection of Coast Kerosene-Type Jet Fuel as the representative jet fuel price is based on two primary reasons. Firstly, it is the predominant aviation fuel used for turbojet and turboprop aircraft. Secondly, the spot commodity price provides a sufficient sample capacity compared to futures prices used in other research due to derivatives eventually expiring.

To ensure the robustness of our findings for out-of-sample data, we partition our dataset into two segments. The first segment covers observations from January 1, 2010 to December 31, 2021 (3014 observations) and is utilized for in-sample distribution fitting including parameter estimation and statistical NHST. The second segment spans from January 1, 2022 to June 30, 2023 (373 observations) and serves as an out-of-sample test specifically designed to compare computational results of risk measures using Matlab [19–23].

### 3.1 Summary statistics of sample indicates a obvious non-normal pattern

**Fig 2:**
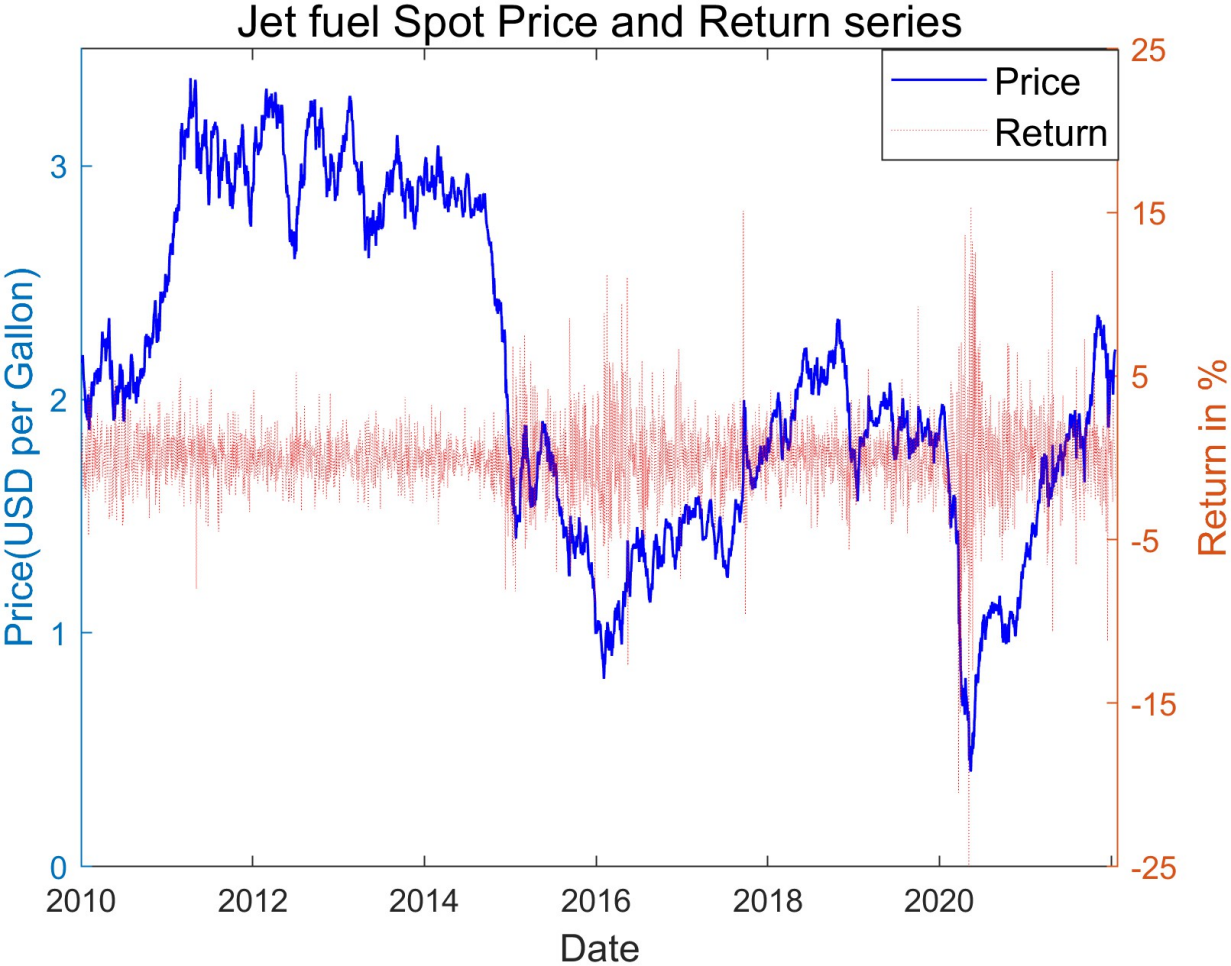
Jet fuel spot price and return data.

Due to the inherent tendency for asset prices to exhibit inflation or non-stationarity, our focus is directed towards the return rates of jet fuel spot price, which can be interpreted as percentage change in price. Both ADF and KPSS test statistics confirm that the return data displays weak stationarity, while the price data does not. The in-sample summary statistics in Table 2 and Fig 2 presented below indicate a non-Gaussian pattern with high kurtosis in the sample statistics of price returns, along with extreme outliers exceeding 6 standard deviations—a phenomenon highly unlikely under normal distribution. Consequently, it is justified to employ a well-founded fat-tail probability model with shape stability to accurately capture the regularity of jet fuel return data.

**Table 2:** Sample summary statistic of price data and return rates.

data	Price(USD/Barrel)	Return rates
mean	1.957	0.00%
median	2.085	0.01%
std	0.693	4.25%
skew	0.105	-0.814
kurtosis	1.931	8.246
max	3.375	14.17%
min	0.407	-24.85%
n	3014	3013

### 3.2 Outperformance in visual distribution fitting and information criterion

**Fig 3:**
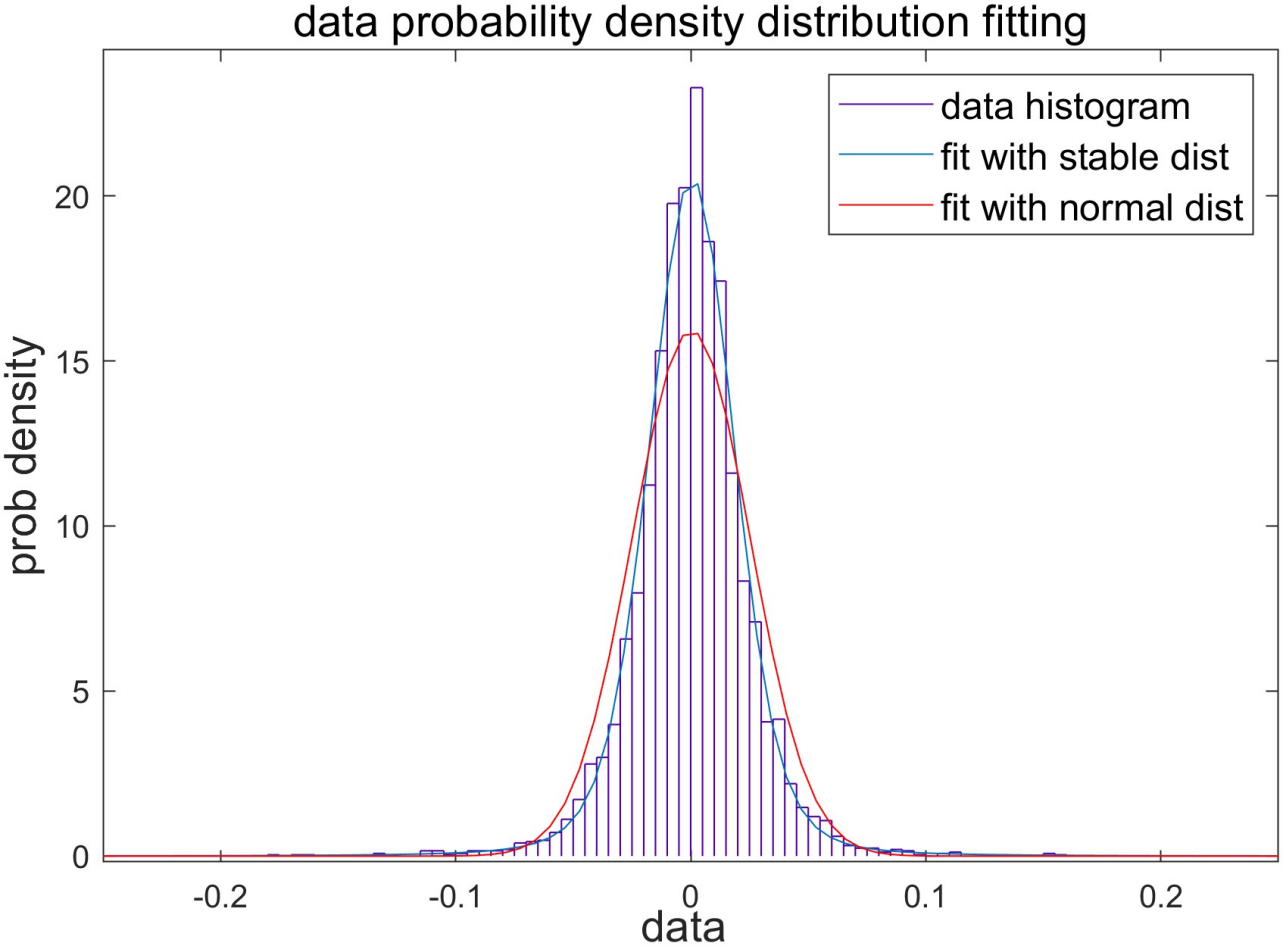
Distribution fitting and parameter estimation.

With the theoretical support of GCLT and the intuitive proof provided by Masaru et al., we performed distribution fitting and parameter estimation based on the 1st sample. As a result, we obtained both probability model parameters in Fig 3.

The histogram displays fitted distributions for stable and normal in blue and red lines, respectively. It is evident that the blue line provides a superior fit. The point estimate of the alpha parameter is 1.63, indicating that the fitted distribution resembles a stable Paretian distribution, as commonly observed in empirical studies due to its heavier tail values and central values with fewer midrange values compared to the normal distribution. Given that stable Paretian models involve more parameters and thus fewer degrees of freedom, it is appropriate to utilize information criteria such as Akaike Information Criterion (AIC) and Bayesian Information Criterion (BIC) to compare the performance of two models while considering penalties for additional parameters. Results from different criteria are provided as Table 3.

**Table 3:** Comparison of information criterion.

criterion\model	normal	stable Paretian
log-likelihood	7051.73	7495.94
AIC	-14099.46	-14983.88
BIC	-14110.98	-15006.92
observations	3014	3014

Based on log-likelihood and both Information Criterion, it is appropriate to conclude that the stable Paretian distribution provides a good fit. To enhance the credibility of this conclusion, we will conduct goodness of fit tests in subsequent sections to further demonstrate that the dynamics of jet fuel returns are more accurately represented by the stable Paretian probability distribution.

### 3.3 Further goodness of fit tests confirm significance in outperformance

As the distribution of data is assumed to be unknown, it is appropriate to employ a non-parametric approach such as the Kolmogorov-Smirnov test to further examine the significance of the outperformance. The findings are detailed in Table 4 for reference.

**Table 4:** Kolmogorov-Smirnov test.

KS test	Stable distribution	Gaussian distribution
stat	0.014	0.083
critval(5%)	0.025	0.025
*p*	0.576	<0.001
sample size	3014	3014
conclusion	fail to reject H_0_	reject H_0_

The *p* of the KS statistics for the in-sample returns, as indicated in the 2nd column of Table 3, is sufficiently small to reject the null hypothesis at both 1% and 5% alpha levels, indicating that the first sample data does not adhere to a Gaussian distribution. However, we are unable to reject the null hypothesis that the data follows a stable distribution given a *p* of 0.57. Additionally, we have conducted a graphical goodness-of-fit analysis to visually and directly demonstrate these differences as shown in Fig 3.

**Fig 4:**
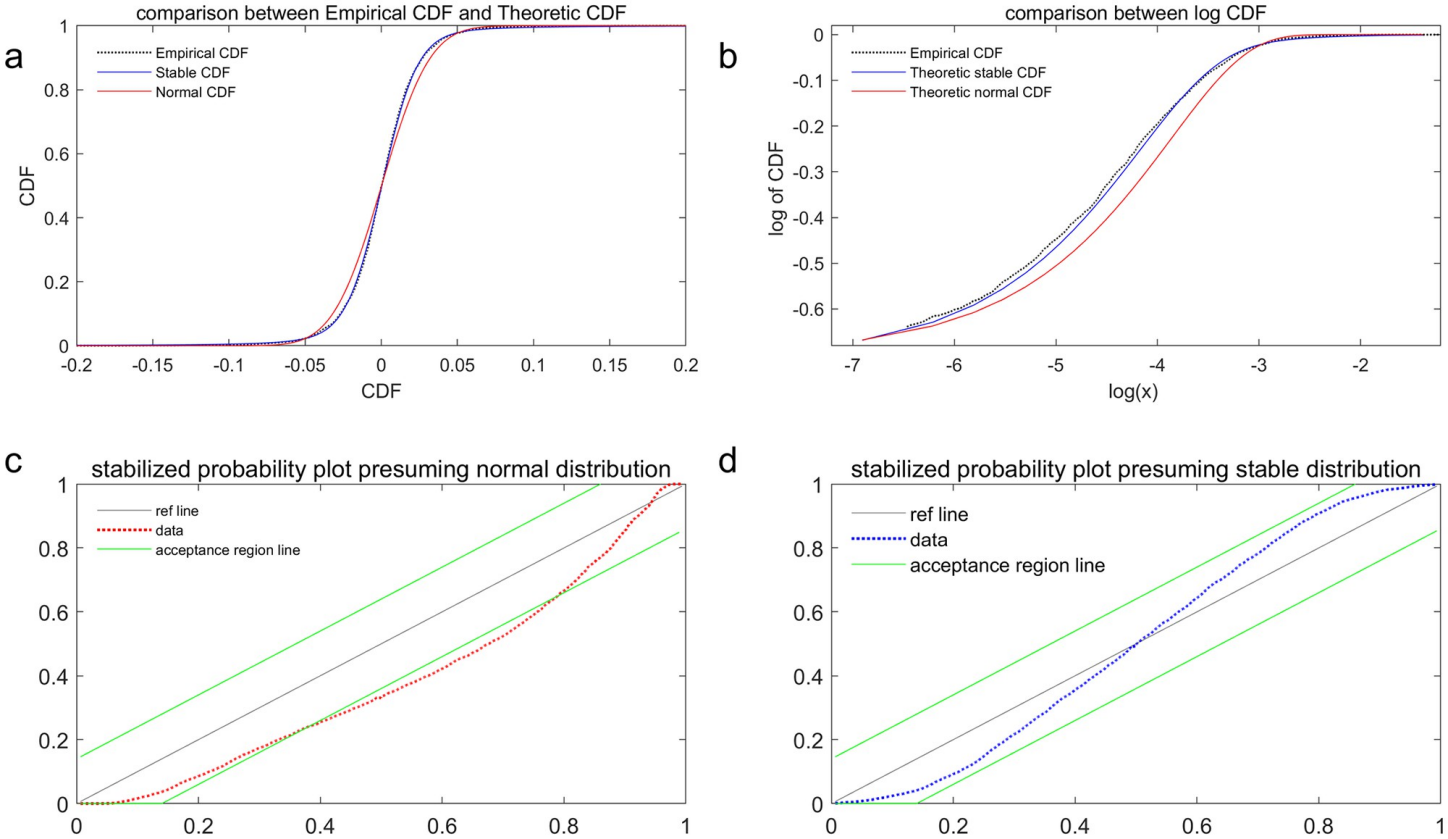
The disparity among the empirical CDF determined by actual data, the theoretical stable Paretian CDF, and the normal CDF.

Fig 4(a) illustrates the disparity among the empirical CDF determined by actual data, the theoretical stable Paretian CDF, and the normal CDF. It is challenging to visually distinguish these CDFs from each other. Therefore, a logarithm transformation of data for x > 0 is performed and presented in Fig 4(b), where the blue line closely aligns with the red line. Fig 4(c) and 4(d) display stabilized probability plots for two probability models respectively. It is evident that all data points fall within the acceptance region under the stable CDF presumption on the right side, while a few points fall outside this region in the left picture. Consequently, it is reasonable to conclude from these tests that a stable distribution indeed provides a better description of the data, justifying further investigation into whether forecasted risk measures demonstrate greater accuracy in computing risk.

### 3.4 Investigate accuracy of risk measures from stable Paretian distribution

We obtain a series of out-of-sample VaR and CVaR values for jet fuel returns from 1 Jan 2021 to 30 Jun 2023using 250-day rolling window setup to visually indicate the differences in model outcomes, The conditional variance is inferred by the GARCH model step-by-step, so risk measures behave differently as the forecast interval becomes wide during volatility clustering. Through cutoff length function mentioned in the work of Vinogradov [40], a symmetric tail truncation is adopted to ensure stable Paretian model has a finite conditional variance that equals to forecast result from GARCH model. Furthermore, discrepancies between these risk predictions and actual losses are tested based on the methodology mentioned in section 2.4.

**Fig 5:**
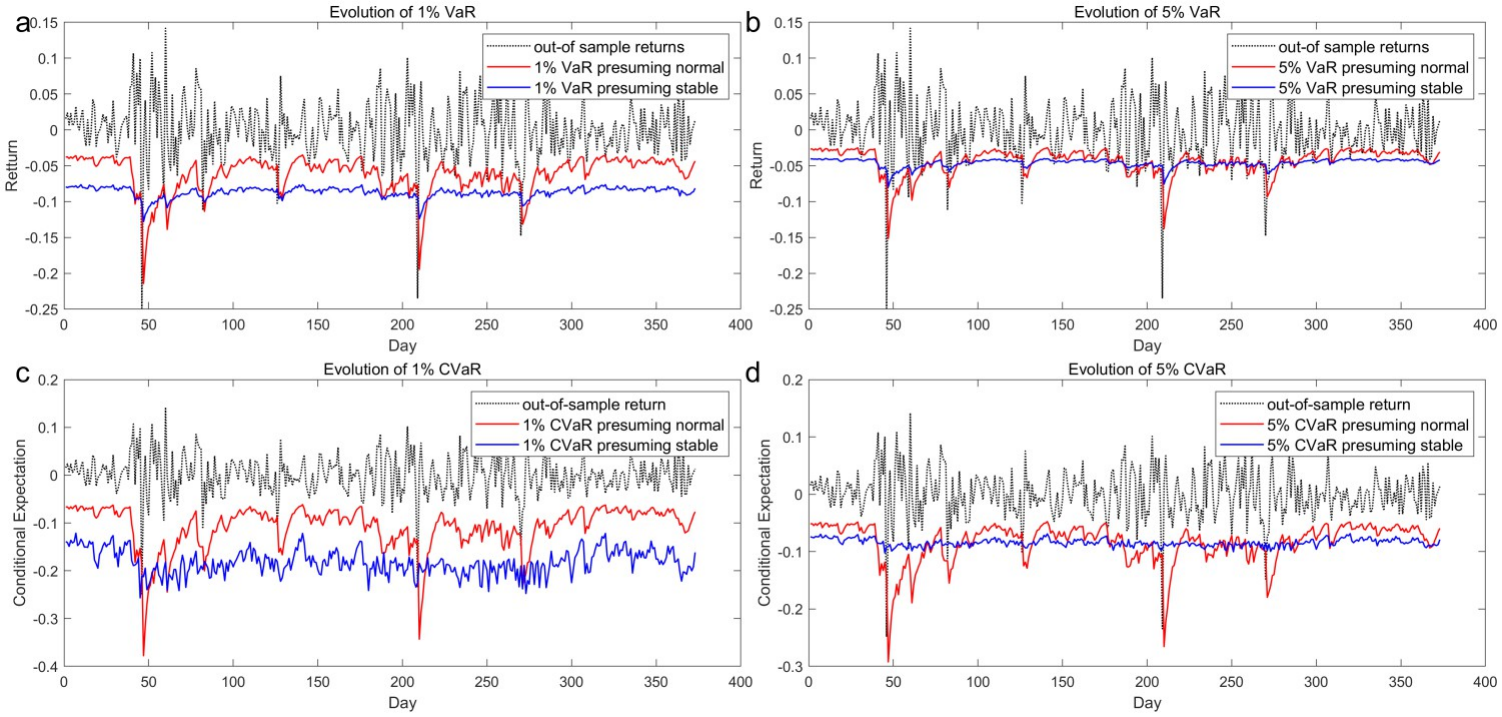
Evolution of rolling window forecast VaR and CVaR.

Three different methods, including the utilization of actual historical data, integration of density functions, and simulation due to the absence of a closed-form expression, are employed for computing risk measures in dynamic situations. Obtaining historical VaR and CVaR from out-of-sample data is a straightforward process after appropriate sorting. Additionally, measurements under the normal model can be directly integrated using the normal density function. However, generating and calculating VaR and CVaR from 1,000,000 simulated random variables following the stable Paretian probability distribution may be time-consuming. Furthermore, discrepancies between these risk predictions and actual losses are tested based on the methodology mentioned in 2.4. We draw the dynamic forecast risk measures in Fig 5 as well as the corresponding test results in Table 5 below.

**Table 5:** The backtesting results of discrepancies of risk measures.

Test name	Test For	Risk measure	Alpha	Result		Test statistic		*P*		Hits	
				normal	stable	normal	stable	normal	stable	normal	stable
Kupiec	Unconditional coverage(Proportion of failures)	VaR	1%	accept	accept	0.019	0.019	0.89	0.89	4	4
			5%	accept	accept	0.024	2.841	0.88	0.09	12	18
Christoffersen	Conditional coverage and independence		1%	accept	accept	0.106	0.106	0.95	0.95	4	4
			5%	accept	accept	1.855	3.642	0.40	0.16	12	18
Dynamic quantile(DQ)	independent of all the past information		1%	accept	accept	3.891	3.133	0.69	0.79	4	4
			5%	accept	accept	4.462	5.066	0.61	0.54	12	18
Strict ESR	Regression coefficients	CVaR	1%	reject	accept	6.567	1.549	0.04	0.46	-	-
			5%	accept	accept	2.074	0.173	0.35	0.92	-	-
Auxiliary ESR	Regression coefficients		1%	reject	accept	6.550	2.525	0.04	0.28	-	-
			5%	accept	accept	2.525	0.177	0.28	0.92	-	-

To show when the loss exceeds thresholds, risk measures here are original negative returns instead of the absolute value. In those figures, the black line represents the actual historical returns from the 2nd sample, while the blue and red lines represent computed risk measures by stable and normal models respectively. Additionally, we tabulate the four types of backtesting results on discrepancies between predicted risk measures and actual losses to facilitate a comparison between the two models. A cursory examination of these results may be frustrating as stable Paretian distribution is less volatile and correlated with actual returns due to evident errors. However, the Gaussian distributed return model gives a more volatile forecast possibly because it is more adaptive to the conditional variance model which was initially constructed based on it. Despite less variation, the stable Paretian model, the blue line, starts with a more conservative prediction which might be capable of better covering loss when dramatic events happen. Meanwhile, there are some clear failures when actual returns move beyond the predicted risk VaR, which is reasonable given our sample size is 373 and confidence levels are 0.01 and 0.05.

In all three VaR test, we can see differences between the two models are so indistinguishable [36, 37]. As Table 4 shows, both models pass the interval coverage test as well as the DQ test though the result of from the stable model under 5% significance levels showing 0.09 p-value in unconditional coverage test, which means a rejection of the null hypothesis under 10% level. However, in the CVaR regression test, the stable model turns out to be superior to that from the Gaussian model under 1% alpha as both regression tests lead to rejection for the latter, which indicates that the regression coefficients are not 0 and 1, meaning a greater bias in risk prediction [39, 41]. The practical significance of risk estimation coincides with the conclusion in static situations that stable Paretian distribution gives a less biased CVaR forecast hence better models of fat-tail risk of jet fuel despite the insignificant underperformance in VaR test. Given the sensitivity of airline corporations to tail risk and the consequences of predictions not covering real losses can be severe, using stable Paretian probability distribution to model the return of jet fuel could be particularly beneficial especially when a hedging strategy to minimize CVaR is considered.

## 4. Conclusions

The majority of expenses in the airline industry are attributed to fuel costs. The increased volatility of jet fuel prices can result in greater uncertainty in cash flow and subsequently higher costs for capital acquisition. To mitigate price fluctuations, many airlines utilize a normal probability distribution assumption to model the stochastic process governing changes in jet fuel prices and guide corresponding hedging operations in accordance with the classical Central Limit Theorem (CLT). In this study, we have innovatively applied a stable Paretian model to fit the return rates of jet fuel data obtained from the U.S. Energy Information Administration, followed by contrasting results using non-parametric goodness-of-fit tests. The superior performance of our model is clearly evident both visually and statistically in the fitting of our distribution, as well as in pairwise comparisons using in-sample data. Additionally, we have assessed the accuracy of computed risk measurements in out-of-sample back tests, and our model demonstrates less bias towards actual numbers. All the aforementioned evidence suggests that computing price risk for jet fuel with a stable Paretian probability distribution, while not perfect, is superior to the common Gaussian probability distribution especially in extreme events. This could be valuable for making risk hedging decisions due to solid theoretical support from GCLT, shape stability, and proper statistical properties with parsimonious parameter numbers.

While this research is limited in its comparison of modeling effects to only two probability models, we believe that the choice to consider normal and stable distributions is justified due to their strong theoretical foundations, favorable statistical properties, and stability in summation. These factors elevate their suitability for application in research to a higher level compared to other probability models. In fact, these two patterns are so commonly observed in empirical data that some scholars have referred to them as "normal" or "stable" laws rather than theorems or models. In our upcoming research, we aim to broaden the scope of data analysis from commodities to publicly traded assets. A promising advancement in cognitive computation of stable models involves adjusting the slope rate at the tails to mitigate the impact of Paretian law. By accelerating the decay rate of the tails, higher moments can be rendered finite, thereby endowing our model with adjustable volatility through selection of different temper parameters. The current trend in research involves combining tempered stable probability distribution with heteroskedasticity processes such as GARCH for dynamic computation of marginal probability distribution with varying conditional mean and variance. These methods enable us to model and compute stochastic processes in a more dynamic and flexible manner, ultimately enhancing decision-making capabilities.
